# Editorial: Emotional impulsivity and emotion regulation deficits as important factors in clinically challenging behaviors in psychiatric disorders, volume II

**DOI:** 10.3389/fpsyt.2026.1969011

**Published:** 2026-09-03

**Authors:** Matthew J. Hoptman, Melissa A. Cyders, Anthony O. Ahmed

**Affiliations:** 1 Clinical Research Division, Nathan S. Kline Institute for Psychiatric Research, Orangeburg, NY, United States; 2 Department of Psychiatry, NYU Grossman School of Medicine, New York, NY, United States; 3 Department of Psychology, Indiana University Indianapolis, Indianapolis, IN, United States; 4 Department of Psychiatry, Weill Cornell Medicine, New York, NY, United States

**Keywords:** challenging behaviors, emotion regulation, psychopathology, treatment implications, urgency

In this Research Topic *“Emotional Impulsivity and Emotion Regulation Deficits as Important Factors in Clinically Challenging Behaviors in Psychiatric Disorders, Volume II,”* we continue our examination of how emotion-related impulsivity and emotion regulation contribute to challenging behaviors. The studies converge on the idea that deficits in regulating emotionally charged behavior influence a number of clinically important outcomes across behavioral, cognitive, neuropsychological, neuroimaging, machine learning, psychiatric genetic, and psychometric approaches. In addition, the diversity of methods and populations illustrates the field’s movement toward integrating mechanisms across levels of analysis, providing a stronger foundation for precision assessment and targeted interventions.

The articles span developmental disorders, community samples, and cross-cultural populations, such as schizophrenia spectrum disorders, anorexia nervosa, grandiose narcissism, and Down syndrome. Rather than examining impulsivity and emotion regulation within isolated disorders, many papers treat them as transdiagnostic mechanisms. The volume addresses outcomes relevant to intervention, including suicidality, risk behaviors, emotion regulation strategies, treatment-seeking intentions, self-stigma, cognitive functioning (including reactive and proactive control, as well as working memory), and developmental factors.

Regarding the latter, Fuca et al. examined the interplay among emotion dysregulation, cognitive function, psychopathological symptoms, and sleep problems in children with Down syndrome (DS). They found that children with DS and emotion dysregulation had poorer visual-motor integration and motor coordination skills, more psychopathological symptoms, and poorer sleep than those without emotion dysregulation. This study highlights the role of emotion dysregulation in the assessment and treatment of DS and the breadth of consequences of emotion dysregulation for such children.

Alghamdi found that exposure to adverse childhood experiences showed a dose-response relationship with psychiatric diagnosis and perceived impact of the diagnosis on their lives. Negative urgency mediated the relationship between adverse childhood experiences and diagnosis, whereas lack of perseverance significantly mediated relationships with diagnosis and with perceived impact. This cross-sectional study provides initial evidence that impulsive traits may be differentially modifiable mechanisms linking early life adversity to adult psychiatric outcomes. Understanding such differential relationships could lead to development and testing of interventions addressing both objective and subjective adult psychiatric outcomes.

Further evaluating the role of impulsivity in risk-taking, Zhang et al. examined impulsivity and emotion regulation self-efficacy across risk-taking domains in Chinese college students. They found positive associations between risk-taking domains and impulsivity, whereas emotion regulation self-efficacy was negatively associated with impulsivity and with health/safety and moral risk-taking. Impulsivity also mediated associations risk-taking and emotion regulation self efficacy, with some differences by sex and risk domains.

Whereas Zhang and colleagues examined a non-clinical young adult sample, Pia et al. examined the contribution of negative urgency, psychiatric symptoms, and neuroimaging measures to suicidal ideation and behaviors (SIBs) in schizophrenia spectrum disorders. LASSO logistic regression identified greater guilt, hostility, and negative urgency as predictors of higher SIB risk, whereas greater difficulty with abstract thinking predicted lower SIB risk. Adding neuroimaging measures improved discrimination between high- and low-risk participants, with greater anterior cingulate thickness and activation across several frontal and cingulate regions associated with lower SIB risk. These findings suggest convergence among emotion-based impulsivity, negative affect, and neural systems supporting cognitive-emotional regulation in understanding suicide risk in schizophrenia spectrum disorders.

Complementing Pia and colleagues’ multilevel approach, Xu et al. examined how emotion regulation strategies and working memory capacity had different effects on proactive vs. reactive cognitive control. They found that cognitive reappraisal correlated significantly with reactive control, whereas working memory capacity correlated with proactive control. These findings highlight differential roles of working memory and emotion regulation in proactive vs. reactive control.

In a study using genome-wide association study data, Bianchi et al. examined genetic correlations between anorexia nervosa (AN) and dimensions of impulsivity. Using genomic structural equation modeling, they found positive genetic correlations between AN and substance use disorder (SUD). However, they found *negative* genetic correlations between AN and both delayed discounting and lack of perseverance, but not other UPPS-P dimensions. These correlations persisted after controlling for SUD. This study highlights the importance of considering how dimensions of impulsivity can inform the genetic dimensions of AN and points to their relevance to diagnosis and treatment of the disorder. More broadly, it highlights the complex role of impulsivity in disorders of constraint.

Moving to treatment engagement, Davis and Drislane examined associations among externalizing proneness, self-stigma, and mental health treatment-seeking in a community sample enriched for mental health issues and impulsivity. Higher self-stigma was associated with greater externalizing proneness, particularly at later self-stigma process, stages, and was weakly associated with lower treatment-seeking intentions. Contrary to expectations, the association between externalizing proneness and treatment-seeking intention was not mediated by self-stigma.

Using a theoretical approach, Raftopoulos and Unterrainer provide an account of affective self-regulation in grandiose narcissism. Integrating psychodynamic theory, contemporary personality psychology, and affective neuroscience, they propose an “envy-contempt spiral” in which upward comparisons that threaten perceived status elicit envy, which is subsequently regulated through contempt and devaluation of others. They conceptualize this dynamic as an affective self-protective mechanism underlying antagonistic behavior and interpersonal conflict

Finally, extending these questions to the measurement of emotion regulation across emotional valence, Larionow et al. studied emotion regulation, specifically cognitive reappraisal and expressive suppression. They found that the Polish language version of the Emotion Regulation Questionnaire – Positive/Negative demonstrated strong psychometric properties and invariance across sexes, setting the stage for continued use of this scale for comparisons across sex. Importantly, this study supported distinguishing between emotional valence in the study of emotion regulation strategies, with cognitive reappraisal of negative emotions emerging as most important. It adds to a growing body of work expanding the study of emotion processes to include positive emotional valence.

This volume reinforces the value of conceptualizing urgency and emotion regulation as multidimensional and transdiagnostic. Across multiple levels of analysis, this volume shows how these processes may shape diverse clinically significant outcomes. Differences across dimensions of impulsivity, regulatory strategies, populations, and outcomes caution against treating either impulsivity or emotion regulation as unitary constructs. Continued integration across levels of analysis, particularly in longitudinal studies, may help identify more precise targets for assessment, prevention, and intervention.

